# Slow protein dynamics probed by time-resolved oscillation crystallography at room temperature

**DOI:** 10.1107/S2052252522009150

**Published:** 2022-09-28

**Authors:** Sylvain Aumonier, Sylvain Engilberge, Nicolas Caramello, David von Stetten, Guillaume Gotthard, Gordon A. Leonard, Christoph Mueller-Dieckmann, Antoine Royant

**Affiliations:** a Structural Biology Group, European Synchrotron Radiation Facility, 71 avenue des Martyrs CS 40220, Grenoble 38043, France; b Hamburg Centre for Ultrafast Imaging, Universität Hamburg, HARBOR, Luruper Chaussee 149, 22761 Hamburg, Germany; c Univ. Grenoble Alpes, CNRS, CEA, Institut de Biologie Structurale (IBS), 71 avenue des Martyrs, Grenoble 38044, France; King’s College London, United Kingdom; University of Padova, Italy

**Keywords:** room-temperature crystallography, time-resolved crystallography, protein dynamics, LOV2 domain of phototropin 2

## Abstract

The development of serial crystallography over the last decade at XFELs and synchrotrons has produced a renaissance in room-temperature macromolecular crystallography (RT-MX), and fostered many technical and methodological breakthroughs designed to study phenomena occurring in proteins on the picosecond-to-second timescale. However, there are components of protein dynamics that occur in much slower regimes, of which the study could readily benefit from state-of-the-art RT-MX. Here, the room-temperature structural study of the relaxation of a reaction intermediate at a synchrotron, exploiting a handful of single crystals, is described. The intermediate in question is formed in microseconds during the photoreaction of the LOV2 domain of phototropin 2 from *Arabidopsis thaliana*, which then decays in minutes. This work monitored its relaxation in the dark using a fast-readout EIGER X 4M detector to record several complete oscillation X-ray diffraction datasets, each of 1.2 s total exposure time, at different time points in the relaxation process. Coupled with *in crystallo* UV–Vis absorption spectroscopy, this RT-MX approach allowed the authors to follow the relaxation of the photoadduct, a thio­ether covalent bond between the chromophore and a cysteine residue. Unexpectedly, the return of the chromophore to its spectroscopic ground state is followed by medium-scale protein rearrangements that trigger a crystal phase transition and hinder the full recovery of the structural ground state of the protein. In addition to suggesting a hitherto unexpected role of a conserved tryptophan residue in the regulation of the photocycle of LOV2, this work provides a basis for performing routine time-resolved protein crystallography experiments at synchrotrons for phenomena occurring on the second-to-hour timescale.

## Abstract 2

The use of room-temperature crystallography to probe protein dynamics on the second-to-minute timescale is demonstrated by monitoring the release of a photoequilibrium induced in crystals of a LOV domain. The slow protein dynamics components include the relaxation of a photoadduct and a crystalline phase transition. The result of the latter is the formation of a non-crystallographic dimer in which the C-termini of the two monomers reorder in distinct conformations on different timescales.

## 1. Introduction

Macromolecular crystallography emerged in the 1950s with the first structure determination of a protein, myoglobin (Kendrew *et al.*, 1958 ▸). At that time, room-temperature macromolecular crystallography (RT-MX), requiring the use of several crystals in order to overcome radiation damage, was the norm until the quest for the structure determination of a ribosome demonstrated the advantage of collecting diffraction data at cryogenic temperature (Hope *et al.*, 1989 ▸). Here, the slowing down of radiation damage meant that complete diffraction datasets could be collected from a single crystal and, as a consequence, RT-MX fell out of favour except for niche experiments such as Laue studies in the context of time-resolved crystallography (Moffat, 2019 ▸) or the investigation of the conformational landscape of enzymes (Fraser *et al.*, 2009 ▸). The development of serial crystallography for time-resolved applications first at XFELs (Schlichting, 2015 ▸), then at synchrotrons (Pearson & Mehrabi, 2020 ▸), triggered a genuine revival of user interest in RT crystallography, which exploited the use of faster detectors (Owen *et al.*, 2014 ▸), the reassessment of both global (Leal *et al.*, 2013 ▸) and specific radiation damage (Gotthard *et al.*, 2019 ▸), and data collections at temperatures well above RT (Doukov *et al.*, 2020 ▸). Taken together, these advances have revitalized the use of RT-MX, particularly to better explore protein dynamics (Fraser *et al.*, 2011 ▸; Woldeyes *et al.*, 2014 ▸; Bhabha *et al.*, 2015 ▸; Russi *et al.*, 2017 ▸).

Phototropins are higher plant blue-light photoreceptors which regulate the response to excessive- or low-light levels, leading to, for instance, phototropism or chloro­plast movement, through two distinct light, oxygen or voltage-sensing (LOV) domains, LOV1 and LOV2 (Christie, 2007 ▸). LOV domains, which belong to the larger Per–Arnt–Sim (PAS) family of small-molecule binding receptors (Taylor & Zhulin, 1999 ▸), non-covalently bind the blue-light absorbing chromophore flavin mononucleotide (FMN). Under blue-light illumination, a covalent bond is formed between FMN and a nearby cysteine residue within microseconds, constituting a photoadduct (Salomon *et al.*, 2000 ▸; Swartz *et al.*, 2001 ▸; Kasahara *et al.*, 2002 ▸). The photoadduct then relaxes in the dark within tens to hundreds of seconds, depending on the nature of the domain (LOV1, LOV2), physicochemical parameters (pH, temperature) and the species (Kasahara *et al.*, 2002 ▸). The formation of the photoadduct is associated with structural changes in the LOV1 and LOV2 domains, most notably the unfolding of the C-terminal helix of LOV2, Jα, which leads to the activation of a serine/threonine kinase domain (Harper *et al.*, 2003 ▸, 2004 ▸).

Due to their light sensitivity, LOV domains have been subject to several kinetic crystallography studies, including at room temperature, as early as 2002 (Crosson & Moffat, 2002 ▸; Fedorov *et al.*, 2003 ▸; Halavaty & Moffat, 2007 ▸). However, these studies focused on the build up of a photoequilibrium within crystals under constant illumination at room temperature, and none were performed in a genuine time-resolved manner. This means that the many orders of time magnitude between the build up of the first intermediate state (in nanoseconds) and the relaxation to the ground state (in minutes) have yet to be characterized with a pump–probe crystallographic approach.

In previous work (Aumonier *et al.*, 2020 ▸), we attempted to characterize, with the shortest time resolution possible, the build up of the photoadduct population within single crystals of the LOV2 domain of phototropin 2 from *Arabidopsis thaliana* (*At*Phot2LOV2, or LOV2 in short). This was achieved by recording full oscillation datasets on a series of 88 single crystals, having first synchronized the start of data collection on the X-ray detector with the illumination of the crystal with a blue LED used for actinic purposes. Time-resolved datasets were constructed by the hierarchical clustering analysis-directed merging of 15-image wedges corresponding to the same 63 ms time segment during the data collection. In this way, we obtained structural snapshots showing the progressive conversion of the resting state population of a crystal into a photostationary equilibrium, reached with a time constant of 1.4 s with the chosen illumination conditions, composed of approximately two thirds photoadduct and one third resting state. This method, termed TR-SOX (time-resolved serial oscillation crystallography) resulted in a time resolution of 63 ms which could, in principle, be increased or decreased by varying the number of merged images.

The large difference in timescale between the build up and decay of the photoadduct (microseconds versus seconds) prompted us to investigate the relaxation of the photostationary equilibrium using a classical crystallography approach, *i.e.* by recording full datasets from single crystals at various time points, up to 20 min after the end of crystal illumination. Combining X-ray crystallography with *in crystallo* UV–Vis absorption spectroscopy, we then monitored the relaxation of the photoadduct population, and obtained similar *in crystallo* time constants from the two methods. Unexpectedly, we observed that, after a time corresponding to twice the relaxation time constant, the space group of the crystals changed from *P*4_3_2_1_2 to *P*2_1_2_1_2_1_, suggesting that the *in crystallo* relaxation of the photoadduct population leads to a phase transition. The change in space-group symmetry is explained by the re-ordering of the C-terminal region of the protein into two distinct conformations in adjacent monomers in the crystal, which is correlated with the flipping of a tryptophan side chain. These results provide a framework for the study of complex protein dynamics occurring on the second-to-hour timescale using classical macromolecular crystallography at room temperature.

## 2. Materials and methods

### 2.1. Protein expression, purification and crystallization

The gene coding for the *At*Phot2LOV2 domain and the transformation protocol were carried out as previously described in our study of photoadduct build up (Aumonier *et al.*, 2020 ▸). However, expression, purification and crystallization protocols were improved compared with those described in our previous work, and are thus briefly detailed here. *At*Phot2LOV2 was produced in *Escherichia coli* BL21 cells grown at 37°C in 2YT medium supplemented with ampicillin at 100 mg ml^−1^ until an optical density at 600 nm of 0.6 to 1.0 was reached. Protein expression was then induced by adding 2 g of arabinose per litre of culture and leaving the cells overnight at 15°C. Cells were then harvested and centrifuged at 4000*g* for 20 min at 4°C. Pellets were resuspended in 20 ml of lysis buffer (100 m*M* Tris pH 7.8, 500 m*M* NaCl, 0.25 mg ml^−1^ lysozyme, 400 mg ml^−1^ DNAse I, 10 m*M* MgSO_4_ and 1 tablet of cOmplete, EDTA-free Protease Inhibitor Cocktail) per litre of centrifuged medium and frozen at −80°C. Thawed pellets were sonicated 5 times 30 s on ice followed by centrifugation at 15 000*g* for 45 min at 4°C. The lysate was loaded on a His-Trap HP 5 ml column (GE HealthCare). Elution was performed with a slow imidazole gradient (10 to 300 m*M* in 25 column volumes). The fractions containing the protein were pooled and dialysed overnight in 20 m*M* TRIS pH 7.8, 100 m*M* NaCl. The protein solution was concentrated at 3 to 4 mg ml^−1^ and loaded on a Superdex 75 10/300 GL (GE HealthCare) size-exclusion chromatography column equilibrated in 20 m*M* Tris pH 7.8 buffer. Eluted fractions were pooled and concentrated to 5 mg ml^−1^. The protein was crystallized in classic 24-well Linbro-style plates with 1 ml per well of mother liquor composed of 100 m*M* MES pH 6.0, 4 to 9% PEG8000 and 50 to 200 m*M* calcium acetate in 2 µl drops (1:1 ratio). Ovoid crystals of ∼100 × 50 × 50 µm^3^ to 200 × 100 × 100 µm^3^ grew in 3 to 7 days at 18°C.

### 2.2. Actinic illumination setup

The same actinic illumination setup [Fig. S1(*a*) of the supporting information] mounted on a cryostream holder was used for both UV–Vis absorption measurements and X-ray data collection [Figs. S1(*b*) and S1(*c*)]. The actinic light, a blue LED emitting at 470 nm (M470F3, Thorlabs) was collimated via a 600 µm fibre to the entrance of a 5× objective positioned 37 mm from the sample position where it produced a 2 mm focal spot. Crystals were mounted in crystal-harvesting loops on the rotation axis of the goniometer, centred in the middle of the focal spot and illuminated for 1 min prior to experimental measurement. With this experimental setup, a 100 × 50 µm^2^ crystal surface received 2.0 µW of actinic light.

### 2.3. UV–Vis absorption spectroscopy

**Figure 1:**
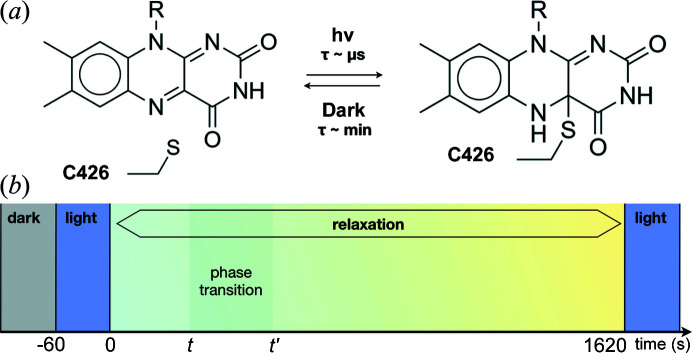
(*a*) Reversible formation upon light irradiation of a covalent thio­ether bond between the sulfur atom from the C426 side chain of LOV2 and the C4a atom of the isoalloxazine ring of the FMN. (*b*) Schematic of the time domains explored in this study. The ground state of LOV2 is probed in the dark (grey). A photostationary equilibrium between the ground state and the photoadduct is built up under steady blue-light illumination for 60 s (blue). Relaxation of the photoadduct is monitored over 1620 s (cyan to yellow gradient). The existence of a crystalline phase transition (light grey) is probed during the relaxation process.

*In crystallo* and solution [Fig. 1 ▸] UV–Vis absorption spectra were recorded at the *ic*OS Laboratory at the European Synchrotron Radiation Facility (ESRF) (von Stetten *et al.*, 2015 ▸) at room temperature using a humidity controller (HC-Lab, Arinax) (Sanchez-Weatherby *et al.*, 2009 ▸) maintained at an experimentally determined humidity level of 98.5% in order to preserve samples from desiccation. The incident white light was provided by a DH2000-BAL lamp (Ocean Optics) connected to the setup via a 200 µm optical fibre and focused at the sample position with a 15× reflective objective. The transmitted light was collimated via the second reflective objective and transmitted via a 400 µm fibre to a QE65Pro spectrophotometer (Ocean Optics). Actinic illumination was provided by a dedicated setup described above [Fig. S1]. Optimal crystal orientation was achieved by rotating the crystal on the goniometer rotation axis until the signal-to-noise ratio of the spectrum was maximal, which implies minimization of the baseline and corresponds to the light path direction as close as possible from the normal of the crystal faces along the smaller dimension. Spectra were recorded every 500 ms after light illumination for a total acquisition time of 5 min. Devices were synchronized using the ESRF OPIOM module. Spectra within a time series were smoothed through a Savitsky–Golay filter using a polynomial function of degree 3 and a window width of 21 data points, then the average absorbance between 600 and 850 nm was subtracted from every spectrum so that each shared the same baseline. An exponential decay model was then fitted on the absorbance at 390 nm from each spectrum in a series. The in-house Python scripts used for this processing are available at https://github.com/ncara/icOS.

### 2.4. X-ray diffraction data collection

All diffraction data were collected at room temperature on beamline ID30A-3 (von Stetten *et al.*, 2020 ▸) at the ESRF. Each crystal was manually mounted on the goniometer and, as for experiments at *ic*OS (see above), the humidity level was maintained at 98.5%. In this study, 27 datasets were collected, each with a 0.3° oscillation range, corresponding to 120° total rotation, for a total exposure time of 1.2 s. The photon flux was ∼1.2 × 10^12^ photons s^−1^ leading to an estimated dose per dataset, as calculated with *RADDOSE-3D* (Zeldin *et al.*, 2013 ▸), reported in Table S1 of the supporting information. Between two and seven individual datasets were collected at distinct positions from a total of eight crystals. The actinic LED was triggered manually from the outside of the experimental hutch via a 5 V pulse generator connected to the LED via the patch panel of the hutch. Delays reported in Table S1 for time-resolved datasets correspond to the time between the end of the actinic illumination and the opening of the X-ray shutter. All the X-ray data collections were performed in the dark. Crystal harvesting and centring were performed under a safe red light.

### 2.5. X-ray diffraction data processing and structure refinement

Diffraction data were processed using the *autoPROC* pipeline (Vonrhein *et al.*, 2011 ▸) and *STARANISO* (Tickle *et al.*, 2018 ▸). Indexing and integration were performed with *XDS* (Kabsch, 2010 ▸) in the two possible space groups (*P*4_3_2_1_2 and *P*2_1_2_1_2_1_) using the ‘-symm’ option in *autoPROC*. The integrated intensities were scaled and merged in *AIMLESS* (Evans & Murshudov, 2013 ▸) and *POINTLESS* (Evans, 2011 ▸) from *CCP4* (Winn *et al.*, 2011 ▸). Dark, photostationary and *R*_xxxx′′_ structures were solved by molecular replacement using *PHASER* with the PDB entry 6qqk (Gotthard *et al.*, 2019 ▸) as the search model. Structures were completed and improved in *Coot* (Emsley *et al.*, 2010 ▸) before refinement with *phenix.refine* (Liebschner *et al.*, 2019 ▸). Models were then optimized through iterative rounds of refinement and model building. The final refinement rounds were carried out with the hydrogen atoms in riding positions to improve the geometry, but were omitted for the final deposition in the Protein Data Bank. Model quality was validated using *MolProbity* (Chen *et al.*, 2010 ▸). Refinement statistics are summarized in Table S2. Figures were prepared with *PyMOL* (version 2.4.0; The *PyMOL* Molecular Graphics System, Schrödinger, LLC). Diffraction images for all 27 datasets have been deposited in the open repository Zenodo under the doi: https://doi.org/10.5281/zenodo.7002447.

## 3. Results

### 3.1. Experimental design

Under blue-light irradiation, the ground state of LOV2 (called D, for dark) is first converted to a short-lived triplet state (called T, for triplet), which is then converted on a microsecond timescale to a long-lived intermediate state (called L, for light) containing a photoadduct formed by a covalent bond between the sulfur atom of a cysteine residue (C426) and the C4a carbon atom of the FMN chromophore [Fig. 1 ▸(*a*)]. Constant light irradiation leads to the build up of a photostationary (PS) equilibrium between the intermediate L-state and a state spectroscopically, but not structurally, resembling the ground state (called D′). Our study aimed to probe the relaxation of the PS equilibrium over the course of tens of minutes with a potential time resolution of ∼1 s. To this end, we applied an identical time scheme to our samples [Fig. 1 ▸(*b*)], corresponding to a certain time period in the ground state (crystal or solution maintained in the dark), 1 min in the PS equilibrium (sample maintained under illumination of a blue LED brought to the sample position by the setup described in Section 2.2) and an ∼30 min time period in the dark, during which the PS equilibrium relaxes to a ground state that is different from ground state D, called D′′. When required, the PS equilibrium was reset at the end of the ∼30 min period with 1 min light irradiation time.

A preliminary round of experiments using a similar experimental design had suggested the existence of a crystalline phase transition occurring between 100 and 200 s after the end of illumination (Aumonier, 2019 ▸). The choice of time points targeted in this study aimed to confirm this hypothesis and understand its cause, which may be related to the relaxation of the photoadduct itself. We first studied the relaxation of the PS equilibrium by UV–Vis absorption spectroscopy in concentrated solutions of LOV2, then in LOV2 crystals, to determine the relaxation time constants. We then carried out structural studies using RT-MX.

### 3.2. Spectroscopic characterization of photoadduct relaxation at room temperature

**Figure 2:**
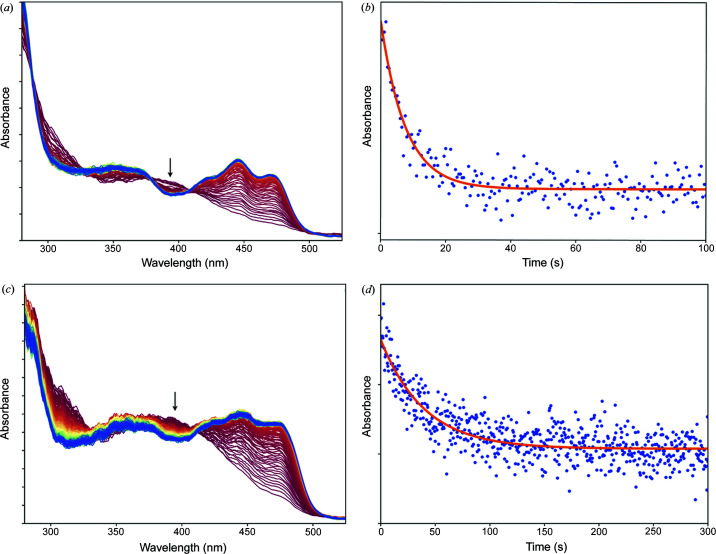
PS equilibrium relaxation monitored by UV–Vis absorption spectroscopy. (*a*) Time-dependent series of spectra recorded at 2 Hz from 0 s (red spectra) to 100 s (blue spectra) after the end of blue-light illumination of a solution of LOV2. (*b*) Time evolution of the absorbance at 390 nm of the solution spectra shown in (*a*), modelled with a monoexponential decay (red curve) with τ = 6.0 s. (*c*) Time-dependent series of spectra recorded at 2 Hz from 0 s (red spectra) to 300 s (blue spectra) after the end of blue-light illumination of a crystal of LOV2. (*d*) Time evolution of the absorbance at 390 nm of the solution spectra shown in (*c*), modelled with a monoexponential decay (red curve) with τ = 40 s.

The ground state D of LOV2 absorbs maximally at 446 and 475 nm both in solution and *in crystallo* (present study), whereas the L-state absorbs maximally at 390 nm (Swartz *et al.*, 2001 ▸). In order to monitor the relaxation of the L-state, we specifically monitored the absorbance decay at 390 nm. In solution samples, relaxation occurs with a time constant of 6.0 s [Figs. 2 ▸(*a*) and 2 ▸(*b*)], whereas in crystals, it occurs around one order of magnitude slower, with a relaxation time constant of 40 s [Figs. 2 ▸(*c*) and 2 ▸(*d*)], suggesting that crystal contacts slow down the formation of the ground state D′′.

### 3.3. Structure determination by RT crystallography of (photo)stationary states (ground and steady states)

Before investigating the time-dependence of photoadduct relaxation and the existence of the crystalline phase transition, we determined the reference structures with the highest resolution possible for (i) the ‘true’ ground state D from unilluminated crystals, (ii) the structural description of the PS equilibrium consisting of a ratio of the L-state and a pseudo ground state D′, and (iii) the ground state D′′ recovered over a time period (1620 s) much longer than both the decay time constant determined by spectroscopy (40 s) and the appearance of the expected crystalline phase transition (<200 s).

#### 3.3.1. Structural differences between the ground-state component D′ of the PS equilibrium and the D-state

**Figure 3:**
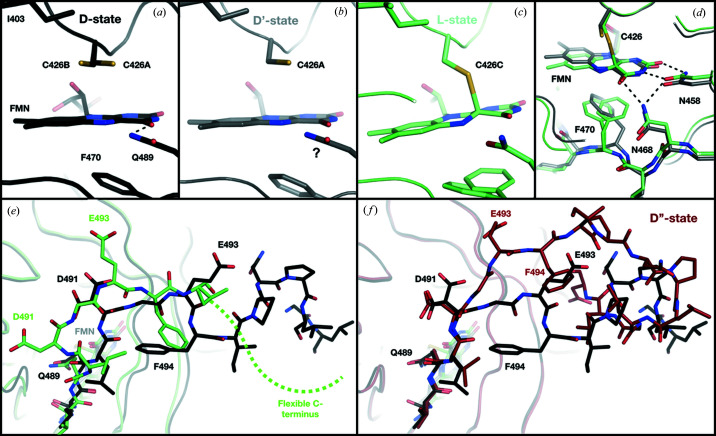
Comparison of the various structures of the ground state and that of the L-state intermediate. Chromophore environments in (*a*) the ‘true’ ground state structure D (carbon atoms in black), (*b*) the ground state structure D′ present in the PS equilibrium (carbon atoms in grey) and (*c*) the intermediate state structure L present in the PS equilibrium (carbon atoms in green). The question mark in panel (*b*) illustrates the fact that the precise orientation of the Q489 side chain in D′ cannot be ascertained from our data. C426 has two conformations in D, and only one in D′. (*d*) Superposition of the D′ and L structures on one side of the chromophore in the PS equilibrium. (*e*) Comparison of the conformation of the C-terminus in D and L. In D, residues 496 to 502 form a short α-helix, which cannot be modelled in either of the structures of the PS equilibrium. (*f*) Comparison of the structure of the C-terminus in D (α-helical) and in one of the two molecules of D′′ (hook-shaped, firebrick).

**Figure 4:**
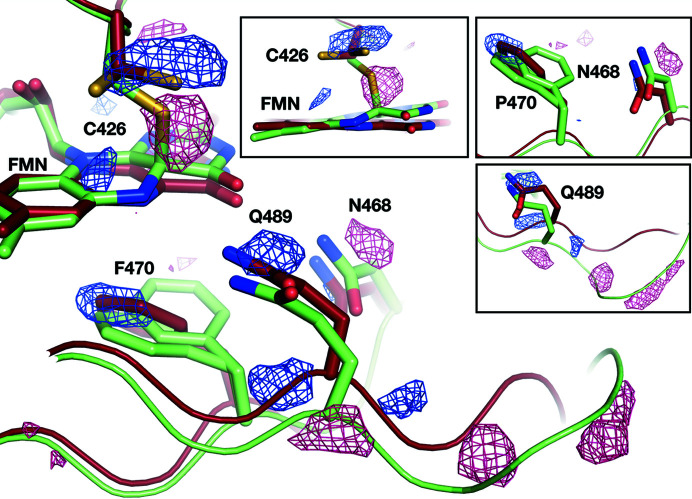
Identification of structural rearrangements around the FMN occurring during the relaxation process. Fourier difference map calculated between *R*_35′′_ and a dataset for the PS equilibrium. Positive and negative peaks are displayed as blue and dark pink meshes, respectively. The L-state (green) and D′′-state (firebrick) models are displayed as ribbons and sticks. Alternate orientations for key residues are shown as insets.

A characteristic of the structure of the ground state D, determined at 1.58 Å resolution at room temperature in the space group *P*4_3_2_1_2 (Table S1), is the presence of two alternate conformations A (major) and B (minor) of the C426 side chain [Fig. 3 ▸(*a*)] and a well ordered C-terminus folded into a short α-helix (Fig. S2). We then recorded datasets under conditions yielding the PS equilibrium, *i.e.* under constant illumination initiated at least 1 min before data collection. The seven resulting datasets were determined in the space group *P*4_3_2_1_2 at a resolution between 1.73 and 2.23 Å. We deposited the best compromise in terms of resolution, data reduction quality and L-state occupancy, which varied between 60 and 90%. The combination of two states (D′ and L) in the crystals made model building somewhat difficult. To facilitate manual rebuilding, and because the D and PS datasets were not isomorphous, we calculated Fourier difference maps between PS datasets and datasets recorded at early time points of the time-resolved series (see thereafter), highlighting very clearly the reorganization of side chains, whole residues and stretches of residues (Fig. 4 ▸). We thus obtained a representative structure of the PS equilibrium, which consists of 60 to 90% L-state structure (see Section 3.3.2) and 10 to 40% pseudo ground D′-state structure, which differs from the D-state structure by the conformation of C426 (one single conformation A versus conformations A and B) [Fig. 3 ▸(*b*)] and a fully disordered C-terminus starting from residue 496 [Fig. 3 ▸(*e*)]. In addition, the precise orientation of the Q489 side chain in D′, which is hydrogen-bonded to a carbonyl group of the FMN in D [Fig. 3 ▸(*a*)], cannot be ascertained from our data [question mark in Fig. 3 ▸(*b*)].

#### 3.3.2. Structural differences between the L-state and the D-state

In the L-state structure, C426 is engaged in a covalent bond with atom C4a of the FMN [Fig. 3 ▸(*c*)]. Our data do not allow us to unambiguously determine the ∼180° rotation of the Q489 head group observed for other LOV domains, but at the minimum, the distance between the Nɛ2 atom of Q489 and the O4 atom of the FMN has increased, supporting the documented loss of the hydrogen bond (Iuliano *et al.*, 2020 ▸). This is coupled with an upward movement of the hydro­philic side of the isoalloxazine ring of the FMN, triggering side-chain rearrangements [Fig. 3 ▸(*d*)] which propagate to the C-terminus, eventually leading to disordering [Fig. 3 ▸(*e*)]. The disordering of the C-terminus is concomitant with a marked reorganization of the N-terminus spearheaded by the reorientation of D491, located at the beginning of the C-terminus (Fig. S3).

#### 3.3.3. Crystal phase transition leading to a different ground state structure D′′

**Figure 5:**
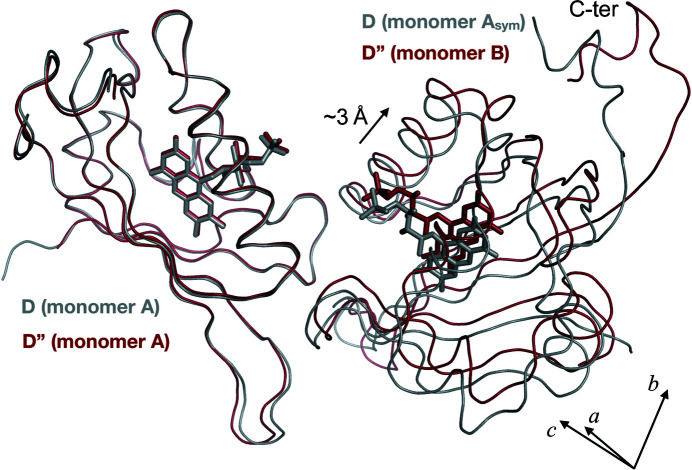
Comparison of the crystallographic dimer from the dark structure D (grey ribbon) with the non-crystallographic dimer from structure D′′ (firebrick ribbon). Both dimers were superposed on their chain A. A translation of ∼3 Å between chain A_sym_ (from D) and chain B (from D′′) along the *b* axis is reported.

While all datasets corresponding to the ground state D and to the PS equilibrium could be indexed and reduced in the space group *P*4_3_2_1_2 with one monomer in the asymmetric unit, the dataset corresponding to the end of the relaxation period we probed (see Fig. S3), and thus to a return to the ground state, can only be indexed and reduced in the space group *P*2_1_2_1_2_1_ with a dimer in the asymmetric unit. In practice, this corresponds to the loss of one symmetry element in the crystal. Both monomers A and B of the dimer show complete absence of the photoadduct, demonstrating that the time point corresponds a new ground state D′′ that is structurally different from D. They differ significantly in the conformation of their C-termini, which, in monomer A, exhibits the α-helical conformation observed in the D structure but, in monomer B, adopts a hook-shaped structure [Figs. 3 ▸(*f*) and S4]. When comparing the crystal packing in both ground state structures, the non-crystallographic dimer of D′′ differs from the crystallographic dimer observed in D by an ∼3 Å translation of chain B, which amounts to a subtle sliding of one molecule compared with the other [Fig. 5 ▸]. This leads to the crystal lattice offering different volumes to the two C-termini of the crystallographic dimer, the larger corresponding to the less structured, hook-shaped C-terminus.

### 3.4. Probing LOV2 dynamics by time-resolved oscillation crystallography

**Figure 6:**
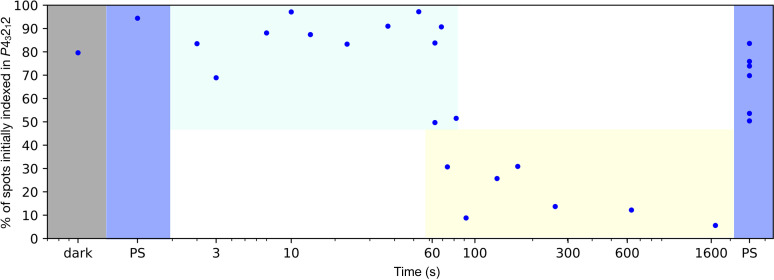
Initial indexing of the various datasets recorded along the illumination scheme. Each dataset was indexed by *autoPROC* with the space group *P*4_3_2_1_2 as input. The percentage of spots indexed in the space group *P*4_3_2_1_2 is represented as a function of time on a logarithmic timescale. All datasets recorded prior to, and during illumination can be confidently indexed in the space group *P*4_3_2_1_2 (>50%). Datasets recorded during the first 60 to 80 s of the relaxation step also show a majority of spots initially indexed in the space group *P*4_3_2_1_2. However, after 60 s, datasets begin to have less than 50% spots initially indexed in the tetragonal space group. For late time points (above 200 s), datasets exhibit an indexing percentage in the space group *P*4_3_2_1_2 below 15% and can only be reliably indexed and reduced in the space group *P*2_1_2_1_2_1_. The fact that all datasets recorded during a second illumination step can be indexed in the space group *P*4_3_2_1_2 demonstrates the reversibility of the space group conversion.

We investigated the time-dependence of photoadduct relaxation and the existence of the crystalline phase transition by recording 1.2 s datasets at various delays after switching off actinic illumination, thus releasing the PS equilibrium. We recorded 18 datasets corresponding to delays of 2, 3, 7, 10, 13, 21, 35, 51, 62 (×2), 67, 72, 80, 90, 130, 166, 258, 630 and 1620 s at resolutions between 1.65 and 2.47 Å. Datasets were named *R*_2′′_, *R*_3′′_, … and *R*_1620′′_. Note that R_1620′′_ corresponds to the D′′-state mentioned in Section 3.3. Data were best reduced in the space group *P*4_3_2_1_2 for all datasets recorded before 67 s and for the dataset recorded at *t* = 80 s (Fig. 6 ▸). Data were best reduced in the space group *P*2_1_2_1_2_1_ for the dataset recorded at *t* = 72 s and for all datasets recorded after 90 s (Fig. 6 ▸). This indicates that the expected crystal phase transition occurs in the 67–80 s time window, significantly earlier and narrower than identified in our preliminary experiments. For comparative analysis purposes, all datasets were eventually reduced in the space group *P*2_1_2_1_2_1_, so as to be able to detect any small differences occurring to either molecules of the dimer in the asymmetric unit at earlier time points.

#### 3.4.1. Photoadduct relaxation

**Figure 7:**
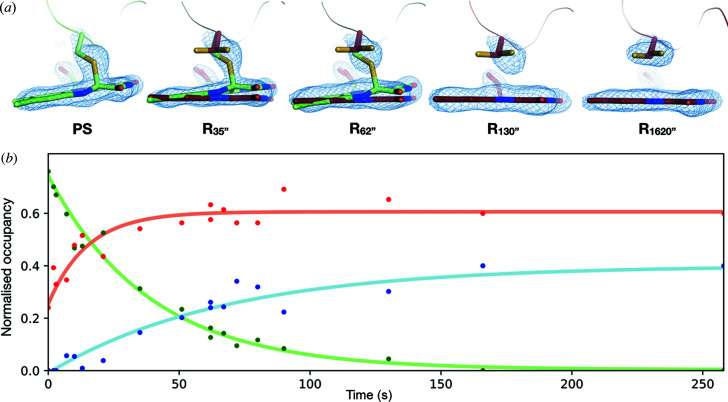
Relaxation of the photoadduct in LOV2 monitored by time-resolved crystallography. (*a*) Evolution of the 2*F*_obs_ – *F*_calc_ electron density map contoured at a 1.5σ level superimposed on the C426 and the FMN chromophore at selected time points between the PS equilibrium (*t* < 0 s) and 1620 s (L-state in green, D′′-state in firebrick). (*b*) Occupancy evolution over time of the three conformations of C426 showing the progressive disappearance of the photoadduct (conformation C: green) and the appearance of the two ground state conformations (major conformation A: orange, minor conformation B: blue).

The variation of unit-cell parameters with time (Table S1) illustrates that datasets at individual time points are not sufficiently isomorphous with others in the series to allow for the calculation of both Fourier difference maps with a high signal-to-noise ratio and reliable extrapolated structure factors. We thus devised a refinement-based method to confidently derive the proportion of the various intermediate states (L-state and D′′-state) at each time point. A series of models were generated with two alternate conformations (one corresponding to the L-state, one to the D′-state) for key residues (I403, I421, C426, L429, I446, F470, Q489) and the FMN chromophore, with respective occupancies varying from 0/100% to 100/0% in steps of 5%. For each of the *R*_xxxx′′_ datasets reduced in the space group *P*2_1_2_1_2_1_, each of these 21 models was subject to molecular replacement, followed by rigid body and individual *B*-factor refinement. *F*_obs_ − *F*_calc_ electron density maps were visually inspected at sigma levels down to 2.0σ to identify the likeliest combination of occupancies (Fig. S5). In a second step, we investigated the presence of the minor conformation of C426 (conformation B), of the α-helical C-terminus observed in monomer A of the D′′-state, the hook-shaped C-terminus observed in monomer B of the D′′-state and the flipping of the W467 side chain. When present, these features were added to the model, which was then subject to positional and *B*-factor refinement. Fig. 7 ▸(*a*) illustrates the evolution of the electron density over the photoadduct during the relaxation from the PS equilibrium to the D′′-state.

The 18 datasets were obtained from distinct locations of six different crystals. The build up of the PS equilibrium in each of these crystals led to variable combinations of L-state/D′-state occupancies. As a consequence, in order to be able to plot the time evolution of occupancies during relaxation, respective occupancies were normalized with respect to the L-state/D′-state occupancies in the PS equilibrium of the crystal on which a given time point was recorded. The occupancy evolution of the various conformations of C426 (A: major conformation in the ground state; B: minor conformation in the ground state; C: photoadduct conformation in the L-state) is plotted in Fig. 7 ▸(*b*). The decay of the C conformation, modelled as an exponential, occurs with a time constant of 39 s, which is in very good agreement with the time constant derived by *in crystallo* UV–Vis absorption spectroscopy (39.6 and 39.7 s for two different time series). The rise of the A conformation occurs significantly faster (time constant of 15 s), which may sound counterintuitive, but can be explained by a rapid plateauing to its maximum occupancy in the ground state, while the appearance of the B conformation slowly develops (time constant of 67 s) and ends up fully compensating the final disappearance of conformer C.

#### 3.4.2. C-terminus reordering

**Figure 8:**
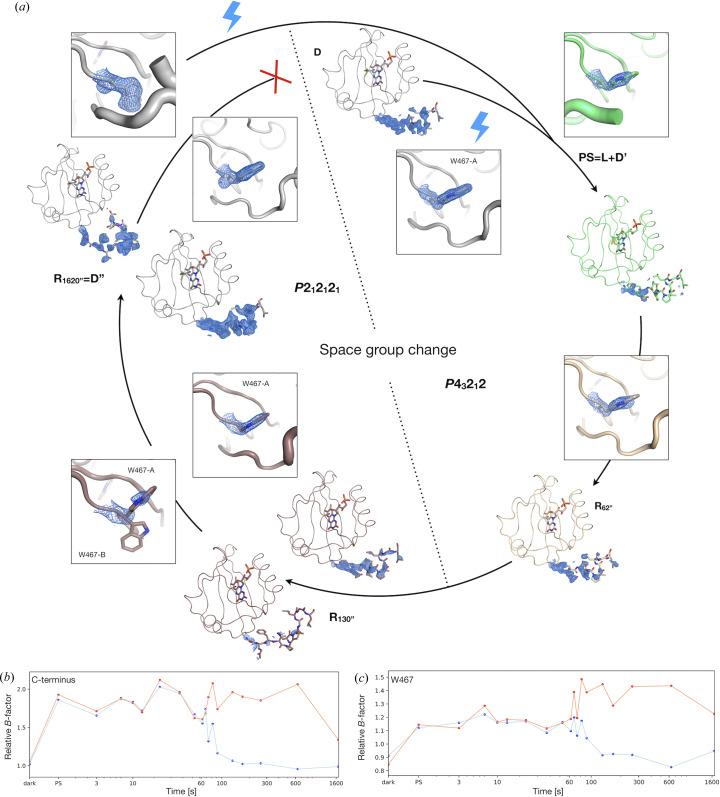
Observation of slow protein dynamics in LOV2 leading to complex C-terminus reordering concomitant with loss of crystal symmetry. (*a*) Structural description of the events occurring to the LOV2 C-terminus along the illumination sequence described in Fig. 1 ▸(*b*). The 2*F*_obs_ − *F*_calc_ electron density map contoured at a 1.5σ level is superimposed on the C-terminus (residues 491 to 503) and on residue W467 for the ground state, then at selected time points between the between the PS equilibrium (*t* < 0 s) and 1620 s. The C-terminus is ordered in the D-state, disorders in the PS equilibrium, then progressively reorders until a crystal phase transition at ∼70 s, at which point each monomer in the asymmetric unit of the new space group orders dissimilarly. One monomer quickly reorders as in the D-state, the other orders more slowly to a distinct, hook-shaped conformation. The phase transition is governed by a flipping of a conserved tryptophan residue positioned in the vicinity of the C-terminus. The resulting photocycle cannot proceed back to the D-state (red cross), but can be reset by re-illumination. (*b*) Illustration of C-terminus disordering/ordering by monitoring of the normalized *B*-factor of the stretch of residues 493 to 495 for monomer A (α-helical C-terminus) in orange, and for monomer B (hook-shaped C-terminus) in blue. (*c*) Corresponding evolution of the normalized *B*-factor of W467, suggesting a rationale for space group transition.

The C-terminus is perfectly ordered up to residue L502 in the dark state D [Fig. 3 ▸(*e*), Fig. S2, start of photocycle in Fig. 8 ▸(*a*)]. It is then fully disordered in the PS equilibrium starting from residue I495, which applies to both the dark state D′ and the L-state, [Fig. 3 ▸(*e*), PS step of photocycle in Fig. 8 ▸(*a*)]. When determining the structures corresponding to the various *R*_xxxx′′_, we already observed the progressive reappearance of electron density on the C-terminus in the early time points, *i.e.* before the crystal phase transition [*R*_62′′_ snapshot of photocycle in Fig. 8 ▸(*a*)]. However, immediately after the transition and the loss of a crystal symmetry element, while one monomer shows an increased level of electron density on the C-terminus (monomer A), the second monomer (monomer B) hardly shows any [*R*_130′′_ snapshot of photocycle in Fig. 8 ▸(*a*)]. Coincidently, the conformation of the C-terminus in the former monomer is that of the α-helical one, and in the latter, of the hook-shaped one. Fig. 8 ▸(*b*) indirectly illustrates the disordering/reordering of the C-termini by plotting the average *B*-factor of residues 493 to 495, normalized by the average *B*-factor of the whole structure. The electron density for monomer B only reappears significantly in snapshot *R*_1620′′_ [Figs. 8 ▸(*a*) and 8(*b*)]. This is in stark contrast with the behaviour of the C-terminus in monomer A which settles before 100′′ [Fig. 8 ▸(*b*)].

The comparison of the C-terminus structure of both monomers allowed us to identify the conformation of the W467 side chain as a key determinant of their respective α-helical or hook-shaped conformations. In the D′′-state, this side chain adopts in monomer A the conformation observed in the D-state (conformation W467-A) whereas in monomer B, it adopts a markedly different conformation, W467-B, where the indole ring is essentially displaced by an ∼90° rotation around the Cα—Cß bond (Fig. S6). In fact, the two conformations are mutually exclusive, *i.e.* one conformation of the side chain sterically prevents stabilization of the opposite C-terminus conformation. Fig. 8 ▸(*c*) illustrates the reorientation of the W467 side chain by plotting its average *B*-factor, normalized by the average *B*-factor of the whole structure. The curves are very similar at early time points but markedly diverge after 70 s. Indeed, W467 has one single conformation at the beginning of the photocycle, which is maintained in monomer A after the phase transition [Fig. 8 ▸(*a*)]. Conversely, the side chain of W467 in monomer B adopts a dynamic equilibrium between 70 and 130 s, which resolves into the single conformation W467-B from then on. Coincidentally, the (70–130 s) time range corresponds to the time period when the percentage of spots initially indexed in space group *P*4_3_2_1_2 hovers around 50% (Fig. 6 ▸).

#### 3.4.3. Resetting the photocycle

The *in crystallo* photocycle described above may appear non-reversible, at least on the timescales we probed (<30 min), since its end point D′′ differs from its starting point D by the conformation of the C-terminus in half the molecules of the crystal. However, we discovered that, by illuminating a crystal in the D′′-state (*i.e.* in the space group *P*2_1_2_1_2_1_), we could restore the PS equilibrium in the space group *P*4_3_2_1_2 (Fig. 6 ▸), bypassing the D-state, and thus reset the photocycle leading to photoadduct relaxation, C-terminus reordering and W467 side chain reorientation [Fig. 8 ▸(*a*)].

## 4. Discussion

In this study, we coupled RT spectroscopy and crystallography to probe the slow protein dynamics occurring on the time scale of tens of seconds to tens of minutes, involved in the relaxation of a blue-light-induced photoadduct in LOV2. *In crystallo*, we could visualize the decay of the photoadduct population following the release of the PS equilibrium, elucidating, under our experimental conditions (crystalline state, composition of the mother liquor, temperature, humidity), a decay time constant of ∼40 s. In addition to this expected result, we observed an unanticipated phase transition, which occurs 70 to 80 s after the release of the PS equilibrium. This phase transition consists of the loss of a crystal symmetry element, which corresponds to the transition of the asymmetric unit from a monomer in the tetragonal space group *P*4_3_2_1_2 to a dimer in the orthorhombic space group *P*2_1_2_1_2_1_. The non-crystallographic dimer is obtained from a crystallographic dimer whose molecules have slid apart. The C-terminus of the protein is fully disordered in the PS equilibrium, then progressively reorders to its α-helical conformation. After the phase transition, the continuation of the ordering of the C-termini develops asymmetrically into distinct conformations and at different rates. While monomer A continues to fold in the α-helical conformation, which is completed within 130 s, monomer B refolds at a slower rate into a very different, hook-shaped conformation, which appears to be fully completed only after 1620 s. The difference in C-terminus conformation is coupled to the orientation of the side chain of a nearby bulky residue, W467, which rotates by ∼90° in order to accommodate the hook-shaped conformation of the C-terminus.

**Figure 9:**
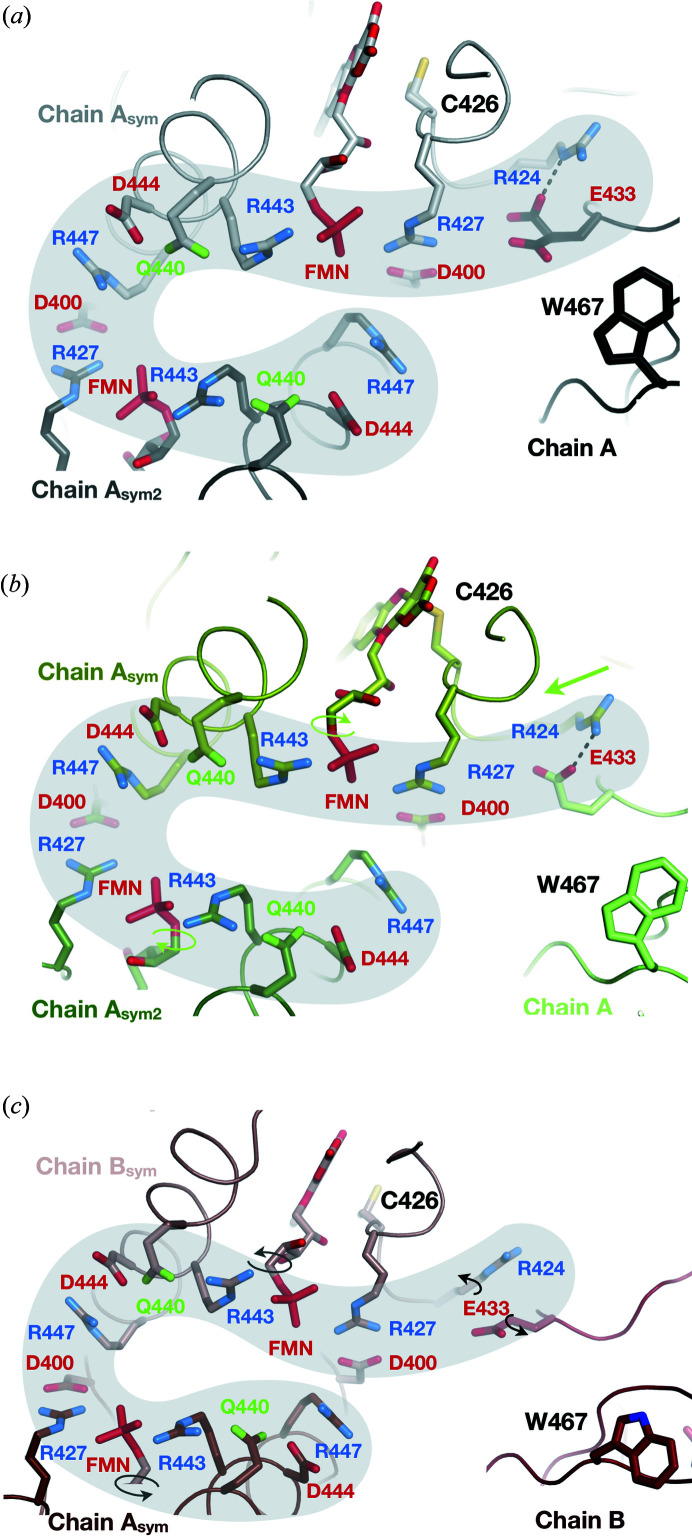
Effect of photoadduct relaxation on the electrostatic network connecting symmetry-related chains. (*a*) The D-state structure is displayed as a black ribbon, and the symmetry-related molecules A_sym_ and A_sym2_ as light-grey and dark-grey ribbons. (*b*) The L-state structure is displayed as a green ribbon, and the symmetry-related molecules A_sym_ and A_sym2_ as split-pea- and forest-coloured ribbons. (*c*) The D′′-state structure is depicted as a firebrick ribbon and the symmetry-related molecules A_sym_ and B_sym_ as dark salmon and chocolate, respectively. Negatively and positively charged residues are depicted in red and blue, respectively, and key residues are represented as sticks. The black dashed line highlights the presence of a salt bridge in the D-state and L-state structures.

The formation of the photoadduct in the PS equilibrium drives the tilt of the isoalloxazine ring of the chromophore, inducing a reorganization of surrounding side chains, which eventually leads to the destabilization of the C-terminus. The release of the PS equilibrium allows the FMN and Q489 to retrieve their original orientation, and residues 489 to 491 to re-stabilize, which then sets up the conditions for C-terminus reordering. The structural origin for the crystal phase transition cannot be definitively ascertained, but the crystal packing analysis of the D-state structure [Fig. 9 ▸(*a*)] reveals a large network of electrostatic interactions between three symmetry-related LOV2 molecules involving eleven charge-bearing residues (seven arginines, one glutamate, four aspartates), two uncharged polar residues (two glutamines) and the negative charges of the phosphate groups of two FMN molecules. The network of interactions is reorganized in the L-state structure on formation of the photoadduct: the movement of the isoalloxazine ring pulls the C426 side chain, which in turn pulls the R426 side chain [straight green arrow in Fig. 9 ▸(*b*)]. Concomitantly, there are rotations of both phosphate groups [circular arrows in Fig. 9 ▸(*b*)]. Relaxation of the photoadduct induces another reorganization of the network of charges [Fig. 9 ▸(*c*)], eventually leading to the breakage of a salt bridge between R424 and E433 belonging to two different LOV2 molecules, which constitutes a key crystal contact in both the D-state and the PS equilibrium in slightly different orientations [dashed line in Figs. 9 ▸(*a*) and 9(*b*)]. The loss of this salt bridge may drive the sliding motion associated with the phase transition. Besides, rotation of both phosphate groups directly influences the position of adjacent residues R427 and R443 altering the charged network around R447 from a symmetry-related LOV2 molecule, thus affecting another crystal contact. Therefore, we propose that the release of crystal contacts associated with the relaxation of the photoadduct induces an asymmetric reorganization of this network of electrostatic interactions, which then drives the sliding motion of the two molecules relative to each other and thus fuels the space group change. As a consequence, the refolding volume available to the C-terminus of each of the two monomers is different, creating possibilities for new types of interactions with neighbouring residues. Most importantly, we observe that residue W467, which belongs to a well conserved FWN sequence of residues found in LOV domains (Fig. S7), acts as a gate discriminating between the two conformations of the C-terminus.

In brief, we observed the appearance of a symmetry-breaking phenomenon during the *in crystallo* relaxation of the LOV2 photoadduct. This is likely not physiologically relevant to this specific LOV domain, as *At*Phot2LOV2 has been decisively shown to be monomeric (Katsura *et al.*, 2009 ▸; Nakasako *et al.*, 2020 ▸) and is thus a probable artefact of the crystal packing that creates non-physiological networks between charged residues and cofactors. However, we cannot exclude that the resulting asymmetric dimer resembles a physiological homodimer or heterodimer formed between other LOV domains. Indeed, the differential C-terminus reordering in the two molecules of a given dimer is reminiscent of a cooperative interaction. Such a cooperative interaction has been described in a heterodimer formed by LOV1 and LOV2 domains within phototropin 2 from *Arabidopsis thaliana*, involved in photoadduct half-life modulation (Kasahara *et al.*, 2002 ▸) and in the conformational changes that lead to J_α_-helix unfolding and kinase domain activation (Oide *et al.*, 2018 ▸). The fact that W467 belongs to a highly conserved short motif can only support this hypothesis.

Our study demonstrates the feasibility of monitoring the progress of protein dynamics at room temperature on timescales that can be considered ‘slow’, from seconds to tens of minutes, using relatively recently developed X-ray crystallography instruments (fast hybrid photon-counting detectors, sample humidity control device). Though our time-resolved approach is based on the quasi-instantaneous activation by light of a photosensitive protein, it can be extended to slower triggers such as ligand diffusion or change in physicochemical parameters (temperature, pH, ionic strength, humidity). The experiments can be carried out using crystals of any size because the timescale of monitoring (seconds to minutes) can be adjusted to well above the timescale of triggering (milliseconds to seconds). One crucial requirement on the bio­logical system is the capacity of the crystalline order to withstand significant structural changes during the time frame of the phenomenon studied. This methodology should greatly expand the prospects of RT crystallography, which comes at a time when X-ray crystallography will, amid the surge of cryo-electron microscopy and deep-learning-based structure prediction tools, likely become more focused on macromolecular dynamics.

## Supplementary Material

Supporting figures and tables. DOI: 10.1107/S2052252522009150/rs5004sup1.pdf

PDB reference: RT structure of LOV2 in a photostationary equilibrium, 8a4e

PDB reference: RT structure of the ground state of LOV2 in space group *P*4_3_2_1_2, 8a2v

PDB reference: RT structure of the ground state of LOV2 in space group *P*2_1_2_1_2_1_, 8a2w

Diffraction images: https://doi.org/10.5281/zenodo.7002447
