# Supplementary material for: Slow protein dynamics probed by time-resolved oscillation crystallography at room temperature
Source: IUCrJ. 2022 Sep 28;9(Pt 6):756–67. doi: 10.1107/S2052252522009150 (PMC9634615; doi:10.1107/S2052252522009150)
Supplement: Supplementary file 1 [file m-09-00756-sup1.pdf]

IUCrJ

Volume 9 (2022)

Supporting information for article:

Slow protein dynamics probed by time-resolved oscillation crystallography at room temperature

Sylvain Aumonier, Sylvain Engilberge, Nicolas Caramello, David von Stetten, Guillaume Gotthard, Gordon A Leonard, Christoph Mueller-Dieckmann and Antoine Royant

Table S1 X-ray data collection and processing statistics

Data set	Dark	PS1	PS2	PS3	PS4	PS5	PS6	PS7	$R_{2"}$
**Data collection and reduction**									
Beamline<br>wavelength (Å)	ID30A-3 (ESRF)<br>0.9677								
Average diffraction-weighted dose (kGy)	169	264	238	162	263	168	203	257	164
Resolution range (Å)	34.77 - 1.58 (1.67 - 1.58)	35.32 - 1.79 (1.97 - 1.79)	39.61 - 2.12 (2.28 - 2.12)	39.66 - 1.96 (2.08 - 1.96)	39.72 - 2.22 (2.33 - 2.22)	39.94 - 1.93 (2.12 - 1.93)	39.77 - 1.73 (1.89 - 1.73)	39.74 - 1.75 (1.82 - 1.75)	39.78 - 2.10 (2.20 - 2.10)
Space group	$P4_32_12$	$P4_32_12$	$P4_32_12$	$P4_32_12$	$P4_32_12$	$P4_32_12$	$P4_32_12$	$P4_32_12$	$P4_32_12$
Unit cell									
a, b, c (Å)	40.82, 40.82, 132.78	41.69, 41.69, 133.07	41.51, 41.51, 132.79	41.55, 41.55, 132.85	41.62, 41.62, 132.72	41.85, 41.85, 133.58	41.68, 41.68, 132.94	41.64, 41.64, 133.08	41.68, 41.68, 133.38
Number of reflections									
Total	112512 (5485)	59573 (3609)	44950 (2027)	64801 (3612)	45355 (2572)	58198 (3954)	71582 (4625)	79583 (3853)	55741 (3033)
Unique	14046 (703)	8624 (432)	5473 (273)	7757 (389)	5478 (275)	6785 (339)	9925 (497)	10869 (543)	6741 (337)
Multiplicity	8.0 (7.8)	6.9 (8.4)	8.2 (7.4)	8.4 (9.3)	8.3 (9.4)	8.6 (11.7)	7.2 (9.3)	7.3 (7.1)	8.3 (9.0)
Completeness ellipsoidal (%)	92.1 (46.9)	91.1 (57.4)	91.1 (66.3)	91.9 (40.6)	89.5 (38.4)	91.9 (68.1)	92.6 (59.0)	88.1 (42.4)	91.9 (40.2)
R_{meas}	0.205 (1.553)	0.138 (1.523)	0.151 (1.601)	0.240 (1.740)	0.234 (2.202)	0.165 (1.471)	0.094 (1.510)	0.113 (1.803)	0.165 (1.704)
R_{pim}	0.071 (0.537)	0.053 (0.521)	0.052 (0.569)	0.082 (0.564)	0.080 (0.711)	0.056 (0.425)	0.035 (0.488)	0.041 (0.654)	0.057 (0.564)
$<I/\sigma(I)>$	6.3 (1.3)	8.3 (1.5)	8.4 (1.4)	5.7 (1.5)	6.6 (1.3)	7.8 (1.6)	11.6 (1.4)	9.8 (1.3)	7.5 (1.4)
CC1/2	0.99 (0.58)	0.99 (0.64)	0.99 (0.64)	0.98 (0.67)	0.99 (0.49)	0.99 (0.78)	0.99 (0.70)	0.99 (0.58)	0.99 (0.49)

Data set	$R_{3''}$	$R_{7''}$	$R_{10''}$	$R_{13''}$	$R_{21''}$	$R_{35''}$	$R_{51''}$	$R_{62''}$	$R_{62''}$
**Data collection and reduction**									
Beamline	ID30A-3 (ESRF)								
wavelength (Å)	0.9677								
Average diffraction-weighted dose (kGy)	166	233	200	267	164	166	200	257	230
Resolution range (Å)	35.77 - 2.01 (2.17 - 2.01)	33.20 - 1.71 (1.80 - 1.71)	35.21 - 1.67 (1.80 - 1.67)	35.20 - 1.87 (2.05 - 1.87)	39.61 - 1.89 (2.12 - 1.89)	39.52 - 1.99 (2.23 - 1.99)	39.58 - 1.85 (2.02 - 1.84)	39.43 - 2.00 (2.14 - 2.00)	39.15 - 1.91 (2.17 - 1.91)
Space group	$P4_32_12$	$P4_32_12$	$P4_32_12$	$P4_32_12$	$P4_32_12$	$P4_32_12$	$P4_32_12$	$P4_32_12$	$P4_32_12$
Unit cell									
a, b, c (Å)	42.21, 42.21, 134.80	41.62, 41.62, 132.80	41.52, 41.52, 132.84	41.52, 41.52, 132.83	41.50, 41.50, 133.10	41.37, 41.37, 133.48	41.45, 41.45, 133.00	41.29, 41.29, 132.89	40.97, 40.97, 132.67
Number of reflections									
Total	50698 (2981)	86466 (4464)	97789 (4567)	61973 (2259)	36495 (2748)	46233 (2605)	61830 (3985)	55241 (2237)	40066 (1395)
Unique	7192 (362)	11891 (596)	11589 (579)	7572 (379)	5877 (295)	5620 (281)	7237 (363)	6459 (323)	5179 (260)
Multiplicity	7.0 (8.2)	7.3 (7.5)	8.4 (7.9)	8.2 (6.0)	6.2 (9.3)	8.2 (9.3)	8.5 (11)	8.6 (6.9)	7.7 (5.4)
Completeness ellipsoidal (%)	92.1 (42.6)	92.9 (42.8)	93.1 (54.5)	91.9 (62.1)	90.2 (73.8)	90.4 (63.5)	92.1 (71.5)	84.8 (39.6)	90.2 (73.2)
R_{meas}	0.575 (1.719)	0.130 (1.187)	0.081 (1.499)	0.130 (1.205)	0.186 (1.408)	0.152 (1.389)	0.084 (1.474)	0.135 (1.466)	0.115 (1.022)
R_{pim}	0.207 (0.600)	0.045 (0.425)	0.028 (0.529)	0.044 (0.479)	0.073 (0.456)	0.052 (0.452)	0.028 (0.438)	0.045 (0.550)	0.040 (0.428)
$<I/\sigma(I)>$	3.4 (1.5)	5.5 (1.5)	12.5 (1.4)	9.1 (1.5)	6.4 (1.7)	8.2 (1.6)	13.0 (1.6)	8.7 (1.4)	9.8 (1.7)
CC1/2	0.89 (0.49)	0.99 (0.87)	0.99 (0.72)	0.99 (0.72)	0.98 (0.73)	0.99 (0.67)	0.99 (0.69)	0.99 (0.51)	0.99 (0.78)

Data set	$R_{67"}$	$R_{72"}$	$R_{80"}$	$R_{90"}$	$R_{130"}$	$R_{166"}$	$R_{258"}$	$R_{630"}$	$R_{1620"}$
**Data collection and reduction**									
Beamline<br>wavelength (Å)	ID30A-3 (ESRF)<br>0.9677								
Average diffraction-weighted dose (kGy)	263	168	169	266	165	284	274	166	164
Resolution range (Å)	38.98 - 2.50 (2.82 - 2.50)	38.76 - 2.21 (2.47 - 2.21)	30.20 - 1.96 (2.25 - 1.96)	40.69 - 2.37 (2.58 - 2.37)	40.95 - 2.48 (2.76 - 2.48)	66.54 - 2.44 (2.67 - 2.44)	40.45 - 2.00 (2.21 - 2.00)	40.89 - 2.01 (2.21 - 2.01)	34.50 - 2.04 (2.17 - 2.04)
Space group	$P4_32_12$	$P2_12_12_1$	$P4_32_12$	$P2_12_12_1$	$P2_12_12_1$	$P2_12_12_1$	$P2_12_12_1$	$P2_12_12_1$	$P2_12_12_1$
Unit cell									
a, b, c (Å)	40.79, 40.79, 132.41	40.51, 42.15, 133.22	41.23, 41.23, 133.07	40.69, 42.17, 133.19	41.05, 42.98, 134.84	40.29, 42.65, 133.08	40.42, 42.47, 132.90	40.22, 42.97, 132.99	40.35, 43.19, 133.10
Number of reflections									
Total	21847 (2012)	34440 (1156)	18406 (870)	31903 (1504)	27749 (1553)	27397 (1356)	36990 (1812)	47968 (1946)	40190 (1996)
Unique	2796 (201)	7737 (388)	4470 (223)	7276 (365)	5967 (298)	6515 (326)	9712 (487)	10198 (511)	10070 (504)
Multiplicity	7.8 (10.0)	4.5 (3.0)	4.1 (3.9)	4.4 (4.1)	4.7 (5.2)	4.2 (4.2)	3.8 (3.7)	4.7 (3.8)	4.0 (4.0)
Completeness ellipsoidal (%)	89.0 (71.0)	85.5 (54.4)	87.5 (67.5)	86.6 (41.9)	87.1 (46.6)	84.6 (35.0)	78.0 (61.8)	80.6 (53.1)	69.7 (29.3)
R_{meas}	0.212 (1.553)	0.126 (0.944)	0.175 (0.807)	0.132 (1.496)	0.202 (1.157)	0.226 (1.302)	0.117 (1.141)	0.193 (1.130)	0.196 (1.262)
R_{pim}	0.076 (0.482)	0.057 (0.518)	0.084 (0.394)	0.060 (0.703)	0.090 (0.495)	0.111 (0.621)	0.058 (0.579)	0.084 (0.561)	0.089 (0.569)
$<I/\sigma(I)>$	6.9 (1.4)	7.3 (1.4)	5.7 (1.8)	8.1 (1.3)	5.7 (1.6)	5.8 (1.4)	7.9 (1.5)	5.6 (1.4)	5.3 (1.6)
CC1/2	0.99 (0.61)	0.99 (0.79)	0.98 (0.84)	0.99 (0.35)	0.99 (0.62)	0.98 (0.41)	0.99 (0.62)	0.98 (0.67)	0.98 (0.49)

Values in parentheses correspond to the highest resolution shell. Average diffraction weighted dose calculated with Raddose-3D

Table S2 X-ray data refinement statistics

Structure	Dark	PS3	D”: $R_{1620”}$
**Structure refinement**			
Resolution range (Å)	30.01 - 1.59 (1.64 - 1.59)	35.23 - 1.96 (2.03 - 1.96)	34.51 - 2.04 (2.11 - 2.04)
Reflections used in refinement	13978 (297)	7749 (114)	10068 (147)
Reflections used for R-free	688 (14)	353 (3)	477 (4)
Solvent content (%)	37	37	37
Number of protein molecules in the a.u.	1	1	2
Number of non-hydrogen atoms			
total	1154	1019	2058
protein	1021	910	1898
Ligands	75	62	62
solvent	90	47	98
Average B-factor			
all atoms	23.76	41.92	42.49
protein	22.72	42.59	42.67
Ligands	21.07	27.93	29.48
solvent	36.82	47.51	47.21
Rwork (%)	16.50	18.89	18.31
Rfree (%)	19.96	22.47	21.40
RMS deviations			
on bond lengths (Å)	0.016	0.016	0.004
on bond angles (°)	1.58	1.38	0.69
Ramachandran			
favoured (%)	99.12	97.14	99.12
allowed (%)	0.88	2.86	0.88
outliers (%)	0.00	0.00	0.00
Rotamer outliers (%)	2.56	0.98	1.44
Clashscore	3.28	5.77	1.55
PDB code	8A2V	8A4E	8A2W

Values in parentheses correspond to the highest resolution shell.

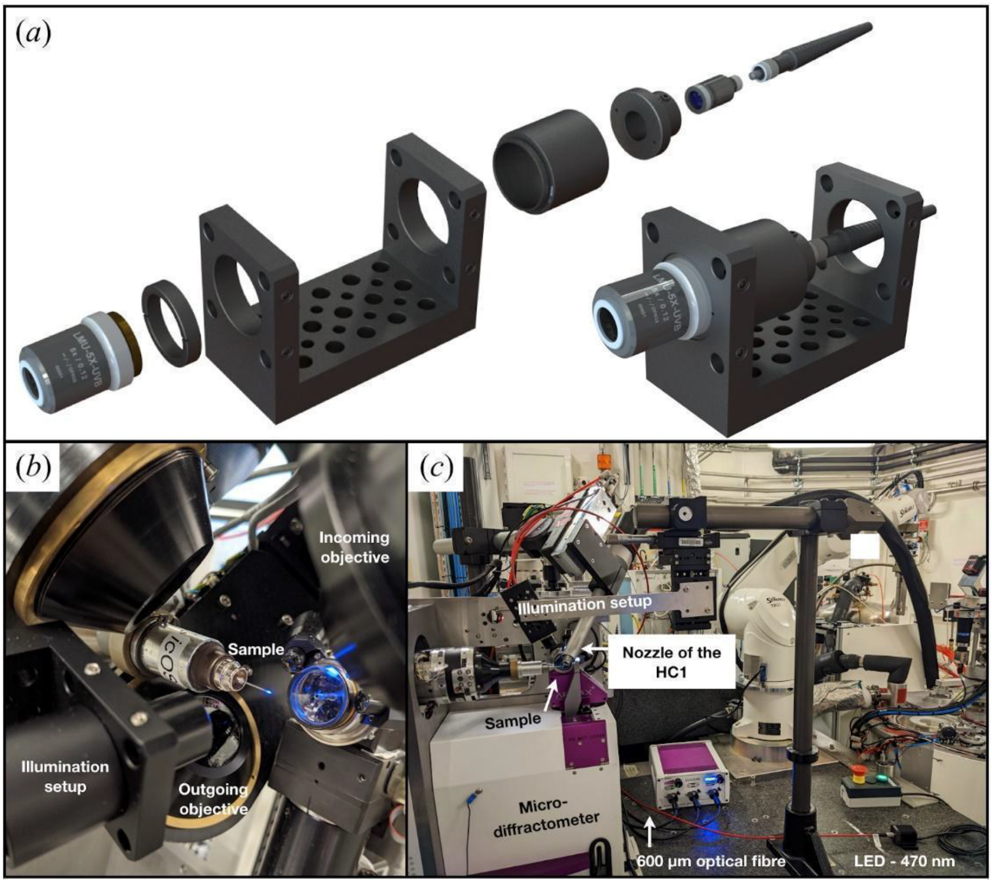

Figure S1 Light irradiation setup. (*a*) Exploded and assembled 3D views showing the design of the setup. (*b*), (*c*) Illumination setup installed at the *ic*OS Lab and on beamline ID30A-3, respectively.

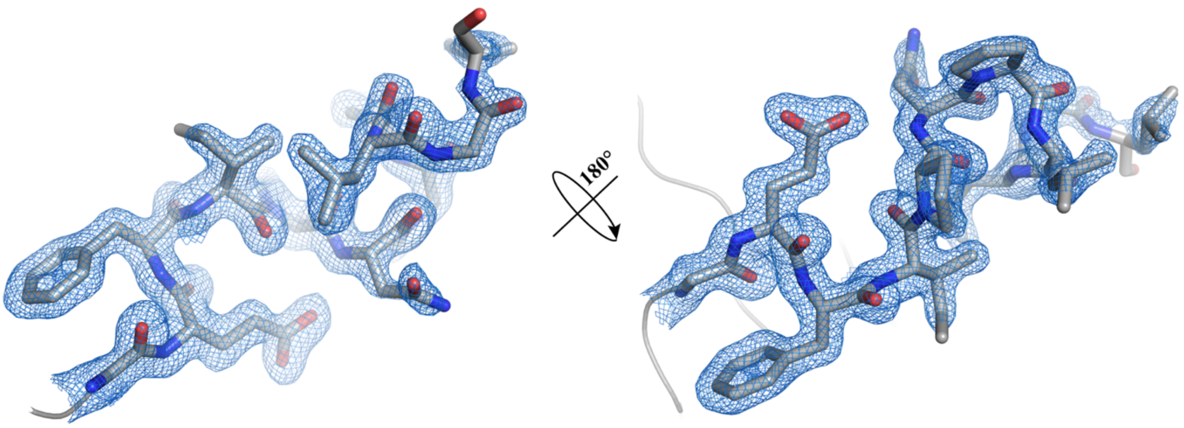

Figure S2 Two orientations of the C-terminus domain of the D-state structure (data set ‘Dark’). $2F_{obs} - F_{calc}$ electron density map contoured at a 1.5 σ level.

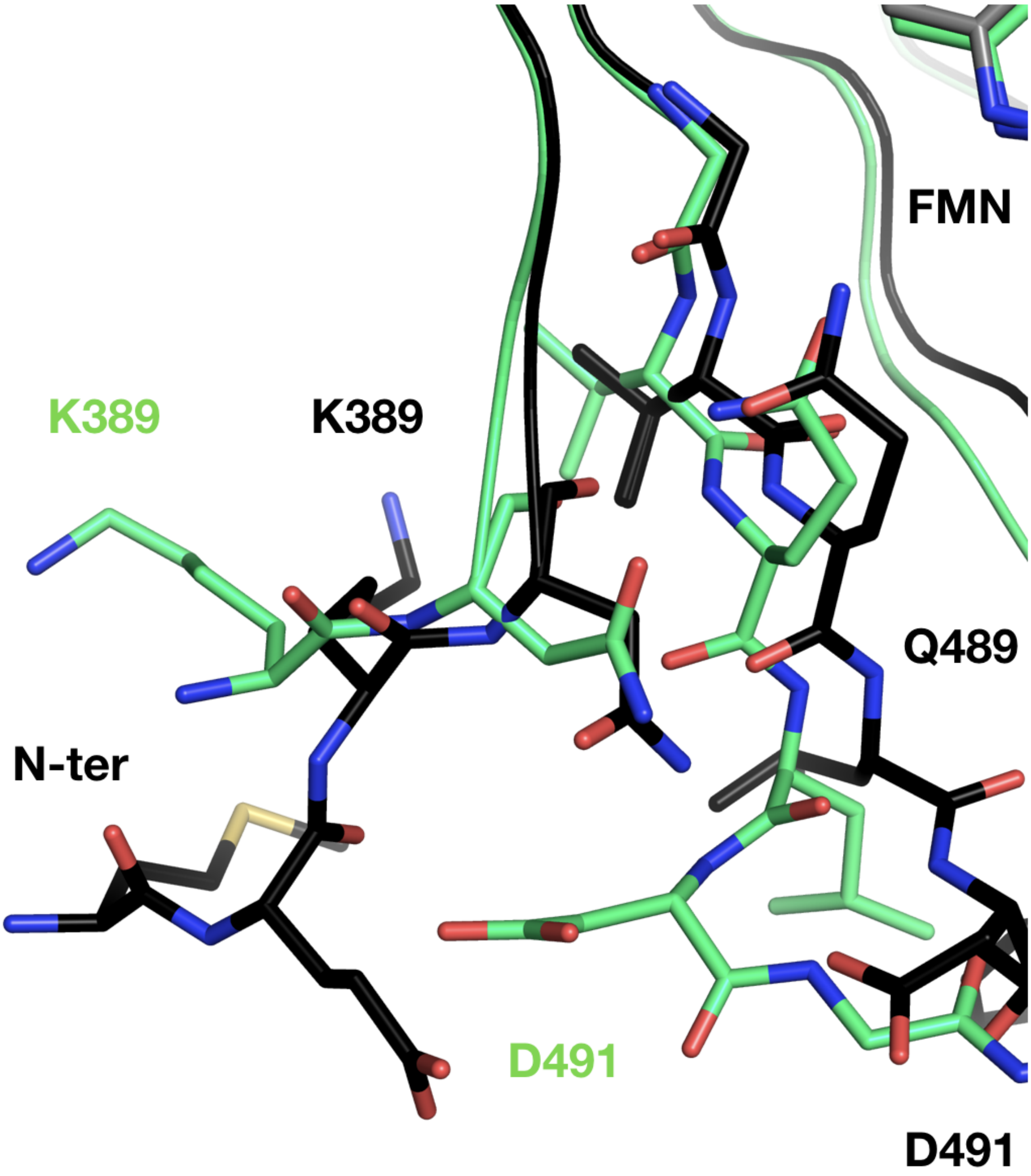

Figure S3 N-terminus reorganisation in the build-up of the PS equilibrium. Superposition of the N-termini regions of the L-state (green sticks) and D-state structure (black sticks).

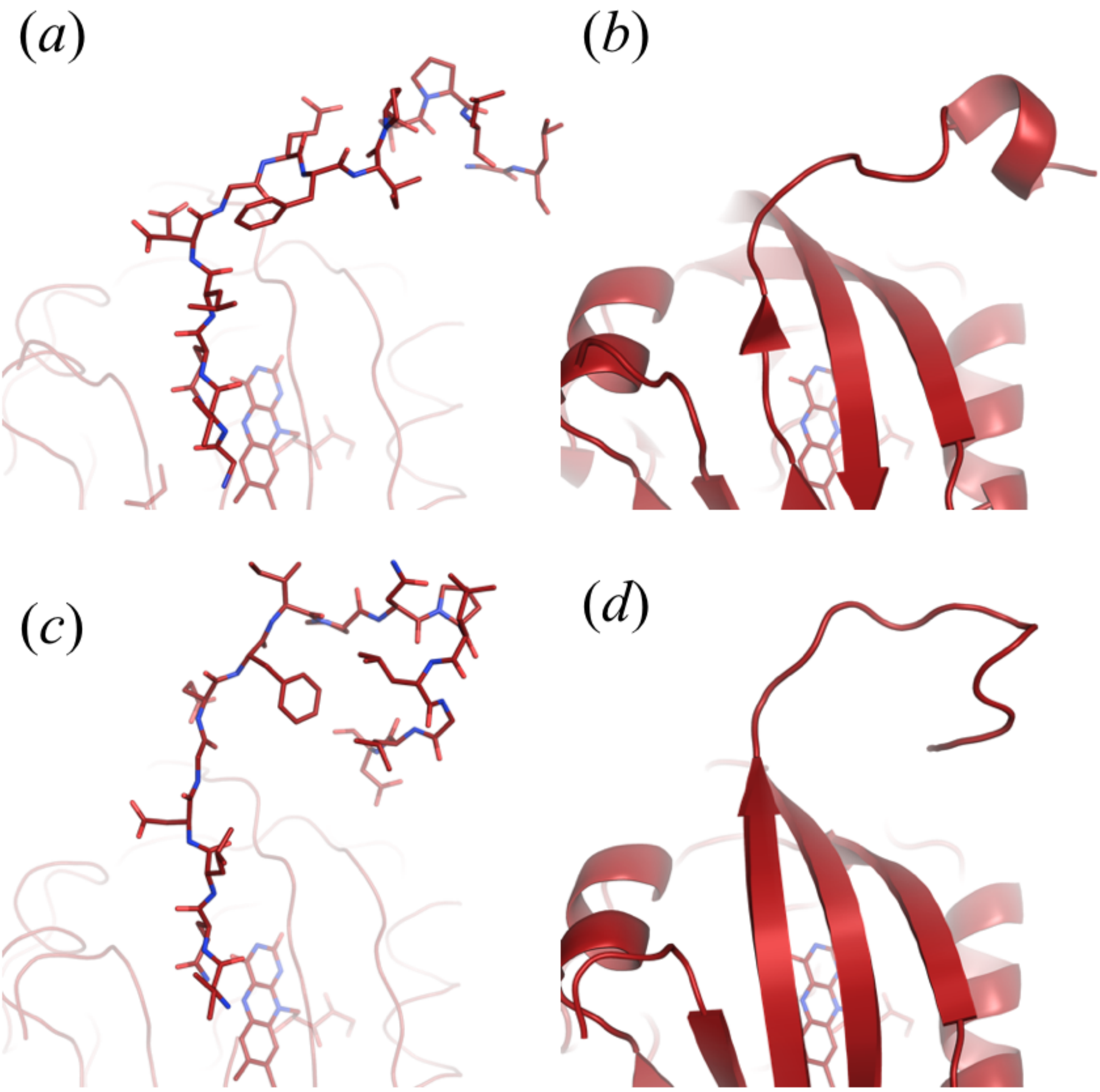

Figure S4 Comparison of the two different C-terminus in the D''-state structure obtained in space group $P2_12_12_1$ with a dimer in the asymmetric unit. The α-helical C-terminus of chain A is shown as sticks and cartoon in panels (*a*) and (*b*), respectively. The hooked-shaped C-terminus of chain B is represented as sticks and cartoon in (*c*) and (*d*), respectively.

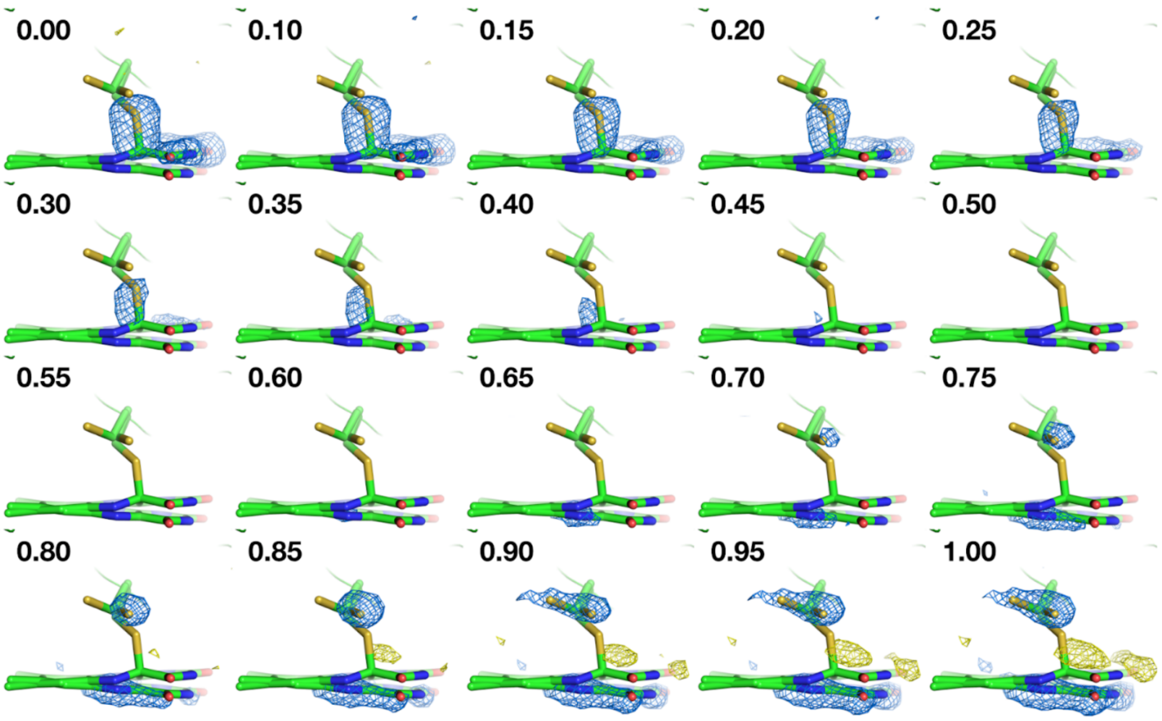

Figure S5 Identification of the adduct occupancy in the $R_{7"}$ dataset. $F_{obs} - F_{calc}$ electron density contoured at a +/-3 σ level (positive in blue and negative in yellow) around the region of the adduct for a series a chimer with adduct occupancies ranging from 0 to 1 by steps of 0.05. The occupancy minimizing difference map peaks here is 0.50.

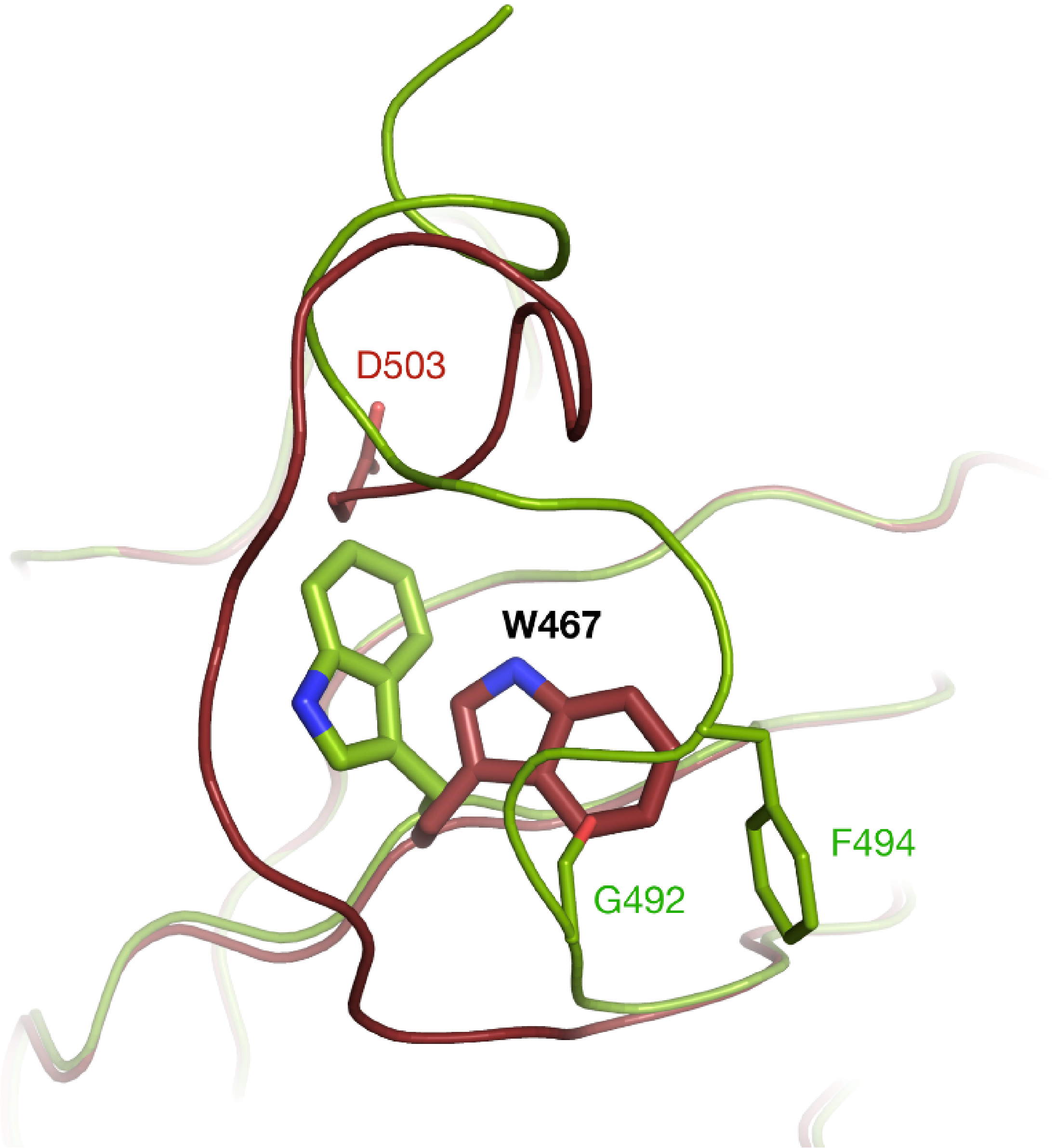

Figure S6 Alternate conformations of W467 in both monomers of the D”-state structure (chain A in green and chain B in ruby) corresponding to the mutually exclusive α-helical and hook-shaped conformations of the C-terminus. W467 and residues involved in steric clashes are highlighted as sticks.

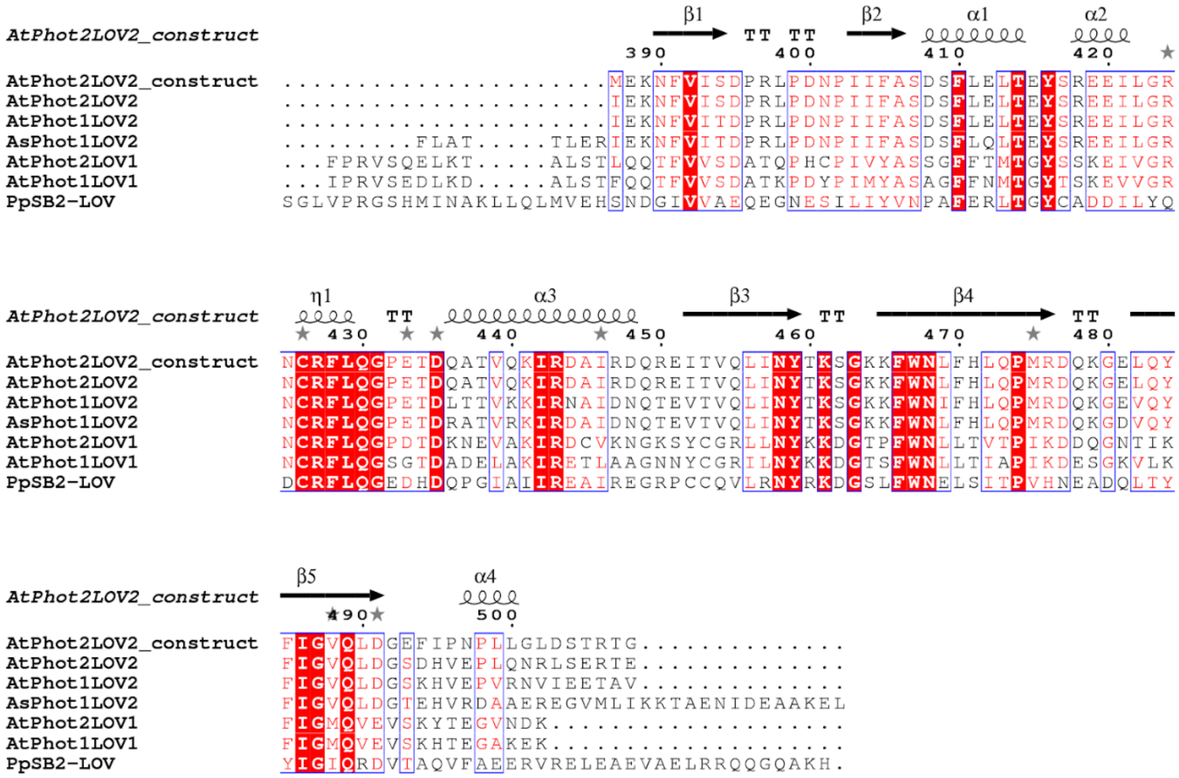

Figure S7 Sequence alignment of various LOV domains from plants and bacteria. Note that the AtPhot2LOV2 sequence featured in our study differs from the physiological sequence after residue 493.
